# The timing of antenatal care access for adolescent pregnancies in Cape Town, South Africa

**DOI:** 10.4102/phcfm.v16i1.4192

**Published:** 2024-02-13

**Authors:** Anja Smith, Grace Leach, Laura Rossouw

**Affiliations:** 1 Department of Economics, Faculty of Economic Management and Business Sciences, Stellenbosch University, Stellenbosch, South Africa; 2 Department of Commerce, Business and Management, Faculty of Economics and Finance, University of the Witwatersrand, Johannesburg, South Africa

**Keywords:** adolescent, pregnancy, maternal mortality, neonate, antenatal care, HIV, South Africa, health inequality

## Abstract

**Background:**

Late antenatal care (ANC)-seeking among pregnant adolescents threatens their health outcomes, and the health outcomes of their new-borns. South Africa has experienced a rapid increase in adolescent pregnancies during the COVID-19 pandemic, adding to the existing concerns around adolescent pregnancy care-seeking behaviour.

**Aim:**

The main aim of this study was to investigate the causes and covariates of late ANC access among adolescents in the Cape Town Metropole, South Africa.

**Setting:**

Three public healthcare facilities in the Cape Town Metropole, 2018–2019.

**Methods:**

A retrospective, cross-sectional study on ANC seeking behaviour was conducted, surveying 202 adolescents. Late attendance was defined as attending ≥ 3 months. For this study, adolescents were defined as women aged 16–18 years. The sample was restricted to adolescents who used public healthcare facilities or who did not attend at all. Data were analysed using univariate, bivariate and multivariate methods.

**Results:**

A total of 50.8% (*n* = 99/195) of the pregnant adolescents in the sample had their first ANC visit > 3 months. 14.9% (*n* = 29/195) did not attend at all. Major contributors to delayed care-seeking include poor pregnancy identification (*n* = 45/99, 45.5%), and a lack of information about ANC. Age, education, and alcohol consumption were significant predictors of delayed care-seeking.

**Conclusion:**

Delayed ANC attendance contributes to negative long-term health outcomes for pregnant adolescents and their new-borns. Improving access to pregnancy tests in the public sector could benefit adolescents with earlier pregnancy identification. Adolescents need to be made aware of their care seeking options.

**Contribution:**

There is evidence of long-term health impacts of late ANC attendance by pregnant adolescents, but there is an absence of evidence on the timing and barriers of late care-seeking behaviour. In this study, late ANC attendance among adolescents was associated with late pregnancy identification and poor knowledge of care options.

## Introduction

Although the global adolescent fertility rate has more than halved, from approximately 86 per 1000 births in 1960 to 42 per 1000 births in 2021 (Adolescent births are measured as births per 1000 women aged between 15 years and 19 years),^1^ it still approximates to 12 million births annually to adolescent mothers (Adolescent births are measured as births per 1000 women aged between 15 years and 19 years).^2^ Childbirth among adolescents significantly increases the risk of maternal mortality because of pregnancy and childbirth complications, especially among adolescents younger than 15 years.^3,4,5^ World Health Organization (WHO) mortality data show that adolescent pregnancies and births are associated with higher risks of eclampsia, puerperal endometritis, and systemic infections than those of slightly older women.^6,7^

In South Africa, the decline in adolescent fertility has plateaued since 2000 and was recorded as 61 births per 1000 female adolescents in 2021, significantly higher than the average rate of 46 in other low- and middle-income countries (LMICs).^1^ Higher teenage fertility rates result in a higher number of births to teenage mothers requiring more timely, appropriate antenatal care (ANC) services as these births risk an array of health consequences for both mother and infant. An increased risk of mother-to-child HIV transmission among adolescent pregnancies has been documented,^8,9^ where adolescent pregnant women are not only less likely to have had a HIV test compared to their older counterparts, but the risk of transmission of HIV between mother and child may be up to three times higher.^10^ This is particularly worrisome in South Africa where approximately one in three women accessing ANC is HIV-positive.^11^

The rate of adolescent childbearing in South Africa is heavily skewed towards socio-economically disadvantaged women: almost 1 in 5 women in the poorest wealth quintile begin childbearing in late adolescence compared with around 1 in 14 for the richest wealth quintile.^12^ In addition, pregnancies among adolescents increased during the 2020 coronavirus disease 2019 (COVID-19) lockdown period. Although national data are not yet available, in Gauteng, the most populous province of South Africa, adolescent pregnancies increased by 60% during 2020.^13^

Children born to adolescent mothers are significantly more likely to be preterm, with low birth weight, and increased likelihood of future stunting.^14,15^ Stunting may result in compromised cognitive functioning^16^ and worse schooling outcomes.^17,18^ There is also evidence of low birth weight children achieving poorer socio-economic outcomes later in life.^19^

Many of the negative health consequences of adolescent pregnancies could be ameliorated by timely and sufficient access to ANC. Appropriate ANC would allow for the detection, monitoring and treatment of treatable maternal danger signs and potential obstetric challenges, beneficial to both neonate and mother.^20^ Late access to ANC for all women, not only adolescents, is positively associated with lower infant birth weight, premature birth, but greater likelihood of infants also needing care in neonatal units after birth, and lower Apgar scores (used to rate the infant’s health at birth).^21,22^ Early ANC also allows for early initiation of antiretroviral treatment, which is associated with better birth outcomes.^23^

World Health Organization guidelines recommend that the first antenatal appointment takes place during the first trimester.^24^ The South African National Department of Health actively monitors access during and after 20 weeks’ gestation as an indicator of timely ANC. The Barometer reports that 30.2% of *all* pregnant women in South Africa sought ANC at a gestational age of 5 months or later.^25^ One study, focusing on a single district, found that almost 65% of adolescent pregnant women attended ANC only after 12 weeks of gestation.^26^ While these studies give some indication of ANC access, there is a dearth of national estimates of ANC timing, specifically for adolescent women.

Timing of adolescent pregnancy care access is not reported as a separate data category in the District Health Information System. While South Africa fares relatively well in terms of overall access to ANC,^25^ less is known about the timing of ANC access, especially adolescent ANC access.

Therefore, the main aim of this study was to investigate the causes and covariates of late ANC access among adolescents. It builds on a similar previous study in metropolitan Cape Town^27^ but adds dimensions specific to adolescents.

## Methods

### Study design

A retrospective, cross-sectional survey on ANC seeking behaviour was conducted. The survey contained mostly closed-ended questions, allowing for quantitative analysis and was adapted from an earlier study. The questionnaire contained sections on socio-demographics, pregnancy discovery, ANC attendance (including reasons for late attendance), the nature of the pregnancy and ANC experiences, including a specific question on the screening tests and services provided at the first full screening visit. Gestational age at first clinic attendance was self-reported. We distinguish between the timing of the first clinic visit and women’s first screening visit.

The data and findings from the study are representative of adolescent mothers who used the public healthcare system to give birth in the Cape Town Metropolitan area in the three sampled facilities. All adolescent mothers aged between 16 years and 18 years of age who used the healthcare facilities to give birth between December 2018 and early April 2019 (from Monday to Saturday) were asked to respond to survey questions given that the day and months of birth are random, this sampling approach allowed us to obtain a simple random sample within the geographic areas covered. A total of 202 women were surveyed.

For this analysis, late ANC access was defined as access after the first trimester. The first trimester corresponds to the WHO definition of timely ANC access.^24,28^ This contrasts with the definition of 5 months (20 weeks) used by the South African Department of Health to monitor timely access for early antiretroviral therapy (ART) initiation purposes.^25,29^

### Study context and population

The study was conducted in three public healthcare facilities in metropolitan Cape Town, in the Western Cape province of South Africa. The Western Cape is one of nine provinces in South Africa and is therefore a subset of the broader South African healthcare system. Each province in South Africa has its own provincial government responsible for healthcare within the province. This provincial autonomy means that the finding from this study may not necessarily apply to the broader South African context.

Two district hospitals, one in the Northern sub-district and the other in Eastern sub-district, and a Midwife Obstetric Unit (MOU) in the Eastern sub-district were chosen. In the South African healthcare system, uncomplicated or lower-risk births are performed in MOUs, while higher risk, assisted natural or caesarean deliveries are performed in district hospitals.

The choice of area was based on the average late ANC access in these areas and builds on the sampling frame of a previous study conducted among non-adolescent mothers (18+).^27^ In the previous study to which this study is a follow-on, healthcare facilities had been selected to focus on geographic areas with high late ANC access rates.

In the Western Cape, it is recommended that pregnant women access ANC prior to 20 weeks of gestation, with a 6-week interval between visits until week 28. The next ANC visit is then scheduled at 34 weeks, and subsequently based on the individual risk profile.^30^ Metropolitan Cape Town was selected because of its relatively poor ANC performance compared with other districts in the Western Cape. In 2018–2019, approximately 66.15% of women presenting at Cape Town facilities did so prior to 20 weeks of gestation.^31^ No distinction is made between adolescent or adult pregnancies in these records.

### Study population and sampling strategy

The selected age category falls outside the WHO definition of adolescents, which ranges from ages 10 years to 19 years.^32^ The sample was limited to adolescents older than 16 because of ethical considerations. The South African Department of Health’s ethical guidelines allows a waiver of parental consent for only children aged 16 and older for sensitive topics such as sexual activity for which parental involvement in data collection may influence their responses.^33^ Ethical clearance for this study was therefore limited to adolescents aged 16–18 years. The upper bound of 18 was chosen as adolescents aged 19 had been interviewed in the same facilities in a previous study and results on this has been reported elsewhere.^27^

Given the relative rarity of adolescent births and administrative report issues with adolescent births and timing of ANC access, it was not possible to accurately power the study sample to be representative of adolescent births in the two sub-districts in which birth facilities were located. The sample can be considered an indicative cross-sectional sample of adolescent births during the relevant period (November 2018 to April 2019) at these facilities. All adolescent mothers who gave birth at these facilities on Mondays to Saturdays were approached by enumerators to be surveyed and almost all agreed to participate in the survey.

Adolescent mothers were surveyed by enumerators using a structured questionnaire (mostly close-ended questions) at the facilities shortly after delivery, at their bedside in the post-labour ward.

Quantitative surveys were conducted between November 2018 and April 2019 following pilot testing at one facility. An amended version of a questionnaire that had been field-tested in a different context in a previous study^34^ was used. An initial sample of five survey interviews were used to pilot the survey and no adjustments were necessary.

The choice to survey mothers at the delivery facility rather than ANC facility was because of the large proportion of adolescent women not attending ANC prior to birth and concerns of losing the most vulnerable from the sample if interviewed after discharge from the facility.

### Data collection: Survey instrument and index variables

The questionnaire was based on the survey instrument used to study late ANC attendance among adult (age 18+) mothers^27^ but extended to include questions on contraception knowledge and use before conception. Gestational age both at first clinic attendance and at first full screening was self-reported.

Given an anticipated low response rate on the household income question, we elected to use an asset index as a measure of socio-economic status (SES).^35^ This was performed by using data on ownership of 14 durable assets by the household collected through the questionnaire. Wealth status was classified according to girls’ and women’s position in the index, classified as either Top (60%) or Bottom (40%).

### Data analysis: Statistical methods

The data were analysed using univariate, bivariate, and multivariate methods. Statistical differences (comparing) in bivariate analysis between women who accessed ANC before or after the first trimester were identified through the chi-square test (χ²) run as a command in Stata. Multivariate analysis of the relationship between late ANC attendance and the selected covariates were estimated through linear probability models using ordinary least square (OLS) analysis. In Appendix 1, we report the results of the analysis drawing on a larger sample which includes respondents who did not attend ANC at all (*n* = 195). *p*-values < 0.05 were considered statistically significant, although *p*-values < 0.1 are also reported but not considered statistically significant for the purpose of this study.

All collected data were captured in SurveyCTO and analysed using Stata 16 (StataCorp [2019] Stata Statistical Software: Release 16. StataCorp LLC, College Station, Texas, United States).

### Ethical considerations

Ethical clearance to conduct this study was obtained from the University of Stellenbosch, Research Ethics Committee: Human Research (Humanities) (No. SU-HSD-002848).

In addition, to ensure that the survey was sensitive to the concerns and circumstances of adolescent experiences, we first conducted focus group discussions (FGD) on the advice of the ethics committee, the results of which are not reported extensively here. At the FGDs, some adolescent mothers from vulnerable backgrounds voiced that once pregnant they do not always receive support from their parents and the broader community. They expressed that their pregnancies bring shame to their families. Some experienced difficulties during their pregnancy, therefore having someone to talk to, even a survey enumerator, would have helped them to voice and process their experiences. Based on the results of the FGDs, the ethics committee approved the study protocol.

Informed consent was obtained prior to survey completion from all respondents, and each respondent received a small gift valued at R25.00 (approximately $1.75) to thank them for their time. All interviews were conducted by enumerators in either English or IsiXhosa. Interviewers were trained to be sensitive to private questions and to treat respondents kindly and respectfully.

## Results

### Health-seeking behaviour and timing of antenatal care access

**FIGURE 1:**
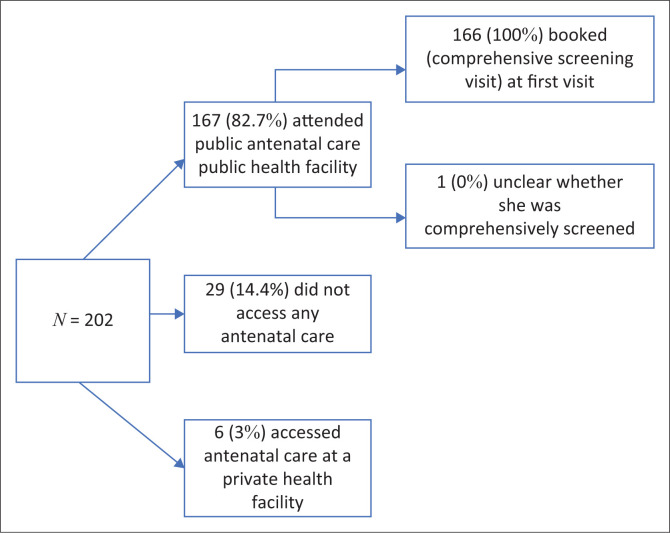
Antenatal care-seeking cascade for respondents.

A total of 202 adolescents provided responses to the survey. Approximately four out of five of the adolescents sampled (*n* = 1677, 82.7%) reported seeking care for their pregnancy at a public healthcare facility (Figure 1), 3% (*n* = 6) of adolescents sought ANC at a private health facility and were therefore excluded from most of the analysis, and 14.4% (*n* = 29) reported not seeking ANC at all. Of the 167 respondents who sought care at a public facility, 166 confirmed that they received a comprehensive screening (aligned with the elements of basic ANC at the time needed to be delivered at the first full ANC visit), while one respondent was unclear and did not provide enough information to be included.

We restricted our analysis to adolescents who attended public ANC clinics and reported full information (*n* = 166) or did not access ANC at all (*n* = 29). Descriptive socio-demographic data on the sample who attended public ANC clinics is provided later in this section.

Most adolescents in our restricted sample (66.15%, *n* = 129/195) reported attending their first ANC visit only after their first trimester (3 months) or not at all (Table 1). While almost 30.0% (*n* = 59) attended their first ANC booking visit between 3 months and 5 months of gestation, more than 20.0% (15.9% + 4.1% + 0.51%) only attended at or after 5 months gestation (Table 1). On an average, adolescents attended 6.6 ANC visits (standard deviation [s.d.] = 2.6) and the average gestational age at their first full ANC visit was 23.6 weeks (s.d. = 12.5).

**TABLE 1:** A summary statistics of timing and frequency of antenatal care visits.

Timing and frequency indicator	*N*	%	Standard deviation
Timing of ANC visit (*n* = 195)			
≤ 3 months	67	34.36	-
3–5 months	59	30.26	-
5–7 months	31	15.90	-
7–8.5 months	8	4.10	-
8.5 months or more	1	0.51	-
Never visited	29	14.87	-
Mean number of visits	6.6	-	2.6
Average gestational age at first full visit (weeks)	23.6	-	12.5

ANC, antenatal care.

**FIGURE 2:**
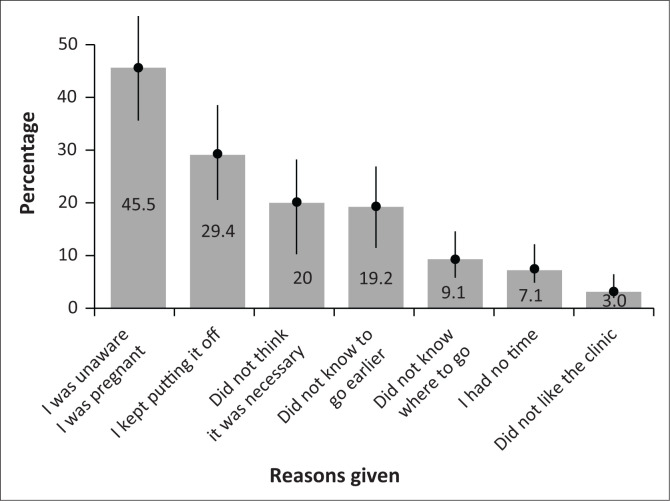
Reasons given by late attenders for not accessing care earlier (*N* = 99).

Among adolescent women who attended ANC late (> 3 months, excluding those who did not attended at all), the majority (45/99 or 45.5%) reported not seeking care earlier because they were unaware, they were pregnant (Figure 2). Another prominent reason includes delaying or putting off the appointment (29.3%). A lack of knowledge and information regarding ANC also contributed to late ANC-seeking behaviour. Among late care seekers, some reported not thinking early care was necessary (20.0%) or required (19.2%) or did not know where to seek care (9.1%).

**FIGURE 3:**
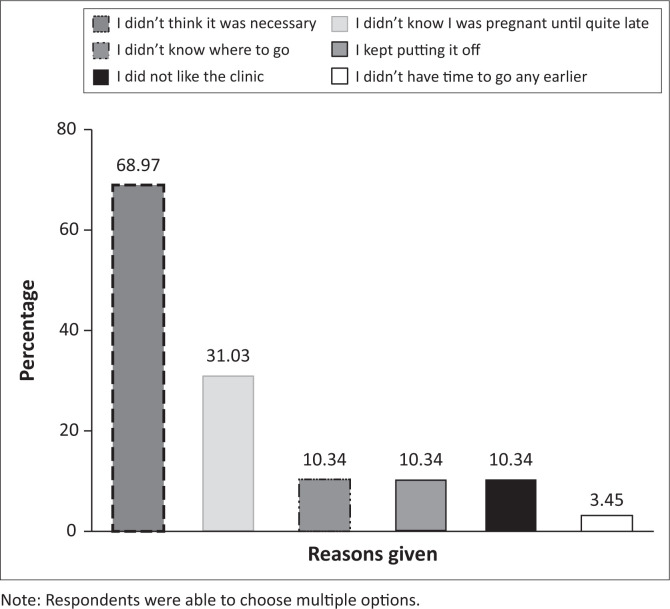
Reasons given by non-attenders for not accessing care earlier (*N* = 29).

Adolescents who attended ANC late (> 3 months) were asked whether there were any personal factors that would have made them go sooner (Figure 3) and overwhelmingly (52.53%) reported that they would have accessed care earlier if they found out they were pregnant sooner. Other prominent factors included if they were told to go sooner (22.22%), if they thought it was necessary (21.21%), or if they thought that their baby had a problem (12.12%).

Adolescents who did not attend ANC at all (*N* = 29) were also questioned (Figure 3). More than two-thirds (68.97%) reported that they did not think it was necessary, while one third (31.3%) of the late attenders reported that they did not know they were pregnant until quite late.

### Socio-demographic and behavioural correlates of late antenatal care access

Table 2 contains the results from the bivariate analysis and multivariate logistics regression results for late ANC attendance (> 3 months), respectively. We distinguish between two sets of results, the characteristics of (1) those who accessed care late (> 3 months) versus early (≤ 3 months), and (2) those who did not access care at all (*N* = 29) and those who accessed public ANC (*N* = 166).

**TABLE 2:** Socio-demographic and behavioural correlates of late antenatal care access.

Determinant	Variable	Early (≤ 3 months)		Late (> 3 months)		*p*	Attended public ANC		Non-attender		*p*	Total (*N* = 195)	%
		*n*	%	*n*	%		*n*	%	*n*	%			
Pregnancy is unplanned and unwanted	No	49	73.13	56	56.57	0.030	105	63.25	2	6.90	0.000	107	54.87
	Yes	18	26.87	43	43.43	-	61	36.75	27	93.10	-	88	45.13
Alcohol consumption	No	60	89.55	74	74.75	0.018	134	80.72	21	72.41	0.307	155	79.49
	Yes	7	10.45	25	25.25	-	32	19.28	8	27.59	-	40	20.51
Employed	No	61	91.04	93	93.94	0.480	154	92.77	26	89.66	0.561	180	92.31
	Yes	6	8.96	6	6.06	-	12	7.23	3	10.34	-	15	7.69
Age (years)	16	16	23.88	27	27.27	0.205	43	25.90	15	51.72	0.003	58	29.74
	17	13	19.40	29	29.29	-	42	25.30	9	31.03	-	51	26.15
	18	38	56.72	43	43.43	-	81	48.80	5	17.24	-	86	44.10
Adolescent is in school	No	33	49.25	46	46.46	0.724	79	47.59	5	17.24	0.002	84	43.08
	Yes	34	50.75	53	53.54	-	87	52.41	24	82.76	-	111	56.92
Behind in school	No	35	52.24	53	53.54	0.870	88	53.01	17	58.62	0.576	105	53.85
	Yes	32	47.76	46	46.46	-	78	46.99	12	41.38	-	90	46.15
Wealth status	Quintile 1	8	11.94	19	19.19	0.514	27	16.27	15	51.72	0.000	42	21.54
	Quintile 2	18	26.87	19	19.19	-	37	22.29	1	3.45	-	38	19.49
	Quintile 3	15	22.39	17	17.17	-	32	19.28	6	20.69	-	38	19.49
	Quintile 4	14	20.90	25	25.25	-	39	23.49	2	6.90	-	41	21.03
	Quintile 5	12	17.91	19	19.19	-	31	18.67	5	17.24	-	36	18.46
Walked to the clinic	No	22	32.84	43	43.43	0.170	-	-	-	-	-	94	48.21
	Yes	45	67.16	56	56.57	-	-	-	-	-	-	101	51.79
Travel time to the facility	Less than 15 min	27	40.30	23	23.23	0.042	-	-	-	-	-	50	30.12
	16–30 min	37	55.22	68	68.69	-	-	-	-	-	-	105	63.25
	31–89 min	2	2.99	8	8.08	-	-	-	-	-	-	10	6.02
	90 min or more	1	1.49	0	0.00	-	-	-	-	-	-	1	0.60
Population group	Black African people	31	46.27	49	49.49	0.637	80	48.19	10	34.48	0.345	90	46.15
	Mixed race people	36	53.73	49	49.49	-	85	51.20	19	65.52	-	104	53.33
	Indian people	0	0.00	1	1.01	-	1	0.60	0	0.00	-	1	0.51
Marital status	Unmarried	52	77.61	81	81.82	0.450	133	80.12	25	86.21	0.320	158	81.03
	Married	11	16.42	10	10.10	-	21	12.65	1	3.45	-	22	11.28
	Living with partner	4	5.97	8	8.08	-	12	7.23	3	10.34	-	15	7.69

ANC, antenatal care.

Respondents were asked if they had planned their current pregnancy and, if not, whether they were happy to be pregnant. Nearly half (45.13%) of the respondents reported that their pregnancy was unintended and that they were unhappy about it. Adolescent women with unintended and unwanted pregnancies were significantly more likely to attend ANC after 3 months as opposed to 3 months or earlier (43.4% vs. 26.9%, *p* = 0.03). Alarmingly, 93.1% of adolescents who did not attend ANC at all reported that their pregnancy was unplanned and that they were unhappy about it. This is significantly (*p* = 0.000) higher than the sample of women who attended public ANC (36.75%).

Approximately 1 in 5 women in our sample self-reported consuming alcohol during their pregnancy. These women were significantly (*p* = 0.018) more likely to attend their first ANC visit after 3 months of gestation (25.25%) than at or before 3 months of gestation (10.45%).

Majority (93.37%) of the adolescents lived within a 30-min travelling distance of the clinics. This figure is skewed towards early ANC attenders. While 40.3% of early attenders lived less than 15 min from a clinic, 23.23% of late attenders lived within the same approximate distance. Among late attenders, 68.69% and 8.08% of adolescents reside within 16 min – 30 min and 31 min – 89 min from their closest clinic, respectively. This is 55.22% and 2.99% for early attenders. The differences are statistically significant (*p* = 0.042).

Women aged 18 were significantly more likely to access ANC early (48.8% vs. 17.24%, *p* = 0.000), while women aged 17 (25.3% vs. 31.3%) and 16 (25.9% vs. 51.72%) were significantly less likely to access ANC. Adolescents who reported being in school were more likely to be non-attenders (52.41% vs. 82.76%, *p* = 0.002). Education is likely to be determined by age among adolescents.

The relationships between late (> 3 months) ANC-seeking behaviour and our range of socio-economic and demographic variables are expressed in Table 3. This analysis only includes respondents who attended public ANC facilities and provided information on our covariates (*N* = 166). The main predictors of late care attendance include pregnancy intention, alcohol consumption and travel mode. Reporting that your pregnancy is unwanted is a significant and positive correlate of late ANC attendance (coefficient = 0.220, *p* < 0.05), even after controlling for covariates (Table 3).

**TABLE 3:** Linear probability model results for women who attended antenatal care ≥ 3 months.

Determinant	Variable	(1)	(2)	(3)	(4)	(5)	(6)	(7)	(8)
Unwanted pregnancy	-	0.172**	0.140**	0.128	0.152*	0.160**	0.198**	0.197**	0.220**
Consumed alcohol	-	-	0.195**	0.190*	0.187**	0.173**	0.107	0.121	0.129
Employed	-	-	-	0.030	0.004	0.019	-0.021	-0.056	-0.082
Age (years)	16	-	-	Reference	Reference	Reference	Reference	Reference	Reference
	17	-	-	0.080	0.091	0.088	0.080	0.068	0.059
	18	-	-	-0.047	-0.084	-0.095	-0.128	-0.132	-0.156
Education	In school	-	-	-	-0.093	-0.092	-0.069	-0.068	-0.039
	Behind in school	-	-	-	-0.035	-0.033	0.012	0.019	0.005
Socio-economic status	Quintile 1	-	-	-	-	Reference	Reference	Reference	Reference
	Quintile 2	-	-	-	-	-0.180	-0.227**	-0.236*	-0.245*
	Quintile 3	-	-	-	-	-0.131	-0.199	-0.206*	-0.213*
	Quintile 4	-	-	-	-	-0.037	-0.100	-0.095	-0.090
	Quintile 5	-	-	-	-	-0.036	-0.154	-0.141	-0.140
Walks to the clinic		-	-	-	-	-	-0.175*	-0.177**	-0.172**
Travel time to the clinic	Less than 15 min	-	-	-	-	-	Reference	Reference	Reference
	16–30 min	-	-	-	-	-	0.162	0.149	0.154
	31–89 min	-	-	-	-	-	0.241	0.212	0.241
	90 min or more	-	-	-	-	-	-0.419***	-0.471***	-0.529***
Population group	Black African people	-	-	-	-	-	-	Reference	Reference
	Mixed race people	-	-	-	-	-	-	-0.074	-0.078
	Indian people	-	-	-	-	-	-	-0.019	-0.051
Marital status	Unmarried	-	-	-	-	-	-	-	-
	Married	-	-	-	-	-	-	-	0.124
	Living with partner	-	-	-	-	-	-	-	0.156
Constant	-	0.533***	0.507***	0.513***	0.588***	0.672***	0.710***	0.760***	0.727***
*R*-squared	-	0.028	0.052	0.063	0.071	0.089	0.152	0.157	0.165
*N*	-	166	166	166	166	166	166	166	166

* , *p* < 0.1;

** , *p* < 0.05;

*** , *p* < 0.01; bootstrapped standard errors.

Similarly, reporting the consumption of alcohol during pregnancy is positively and significantly associated with late ANC attendance (coefficient = 0.173, *p* < 0.05), but the variable is insignificant in a multivariate regression (see columns six to eight of Table 3). Adolescents from wealthier households are less likely to access ANC late, but this difference is only significant at a 10% level where we observe the difference in behaviour among those in quintile 2 (coefficient = −0.245, *p* < 0.1) and quintile 3 (coefficient = −0.213, *p* < 0.1) compared with those in quintile 1 (see column eight of Table 3). Adolescents who reported that their mode of transport to the health facility was walking were significantly less likely to access care late (coefficient = −0.172, *p* < 0.05) (see column eight of Table 3).

In the appendix (Appendix 1), we report the results of the analysis drawing on a larger sample, which includes respondents who did not attend ANC at all (*n* = 195), opposed to the 166 respondents in Table 3. The dependent variable in these regressions is equal to one if the adolescent attended ANC late or not at all. In these new multivariate analyses, we include a binary variable controlling for non-attendance, and exclude travel mode and time as this was not captured for non-attenders. The results for this larger sample remain similar in size and significance. Reporting that a pregnancy was unwanted (coefficient = 0.158, *p* < 0.05) and reporting the consumption of alcohol during pregnancy (coefficient = 0.145, *p* < 0.05) are significant and positive correlates of late ANC attendance (Additional File 1).

## Discussion

Almost two thirds (66.15%), of pregnant adolescents sampled had their first ANC booking after 3 months of gestation or did not attend ANC at all and the average gestational age at their first full ANC visit was 23.6 weeks, which is later than the cut-off of 20 weeks gestation monitored by the National Department of Health. Women who accessed care after 3 months would have been too late to have received termination of pregnancy services from a primary health care facility if this was their chosen option.^36^

Major contributors to delayed care-seeking among late attenders include poor pregnancy identification (*n* = 45/99, 45.5%), and a lack of information about ANC, including not thinking early care was necessary (*n* = 20/99, 20.0%) or required (*n* = 19/99, 19.2%), or did not know where to seek care (*n* = 9/99, 9.1%). Age, education, and alcohol consumption were significant predictors of delayed care-seeking.

One of the key self-reported reasons for delayed care-seeking behaviour (45.5%) is not knowing that they were pregnant, and half of the adolescents reported that they would have gone earlier if they found out they were pregnant sooner. There are multiple reasons that could explain poor pregnancy identification, including a lack of knowledge about the signs of pregnancy or biological or behavioural reasons.^27^

Late care-seeking behaviour was often driven by delayed or deliberate postponement of the appointment (29.3%). Our study provides quantitative evidence of the proportion (magnitude) of adolescents who delayed or deliberately postponed their appointment. A small, qualitative study at an urban Western Cape MOU found that some of the additional barriers include fear of disclosing their pregnancy to their family until they are unable to hide it; shock, fear and potentially denial and negative perceptions of how they might be treated at the healthcare facilities.^37^

In our sample, a lack of knowledge and information around early ANC and its benefits is evident, specifically not knowing that it is necessary (20.0%) or required (19.2%). A few respondents also did not know where to seek care. The number of adolescents who did not attend any form of ANC was unexpectedly large (15.0%), with a major barrier among this group being that they did not know it was necessary (69.0%).

In other smaller studies on adolescent pregnancies in South Africa, supply-side factors related to the clinic or healthcare workers like fear of verbal aggression or poor communication from staff^38,39^ and long waiting times at clinics^39^ deterred access. This was not the case in our sample, where only 3% of late accessors reported not liking their clinic as a reason. This contrasts with the findings from other studies.

In our study, demand-side factors appear to be driving late ANC-seeking behaviour among adolescent pregnancies. One of the strongest predictors of late access is pregnancy intention, where almost half of the adolescents sampled reported that their pregnancy was unintended and unwanted.

A strong predictor of late care-seeking behaviour is whether an adolescent self-reported consuming alcohol, which may act as a proxy for risky behaviour. Similar results have been found among non-adolescents.^27^ Age and education were significant predictors of ANC timing, as adolescents with Grade 12 education were significantly more likely to seek care before as opposed to after 3 months (*p* = 0.027). Education is often associated with ANC utilisation through its influence on the perceived need for care and increased confidence in making health decisions.^40^

The lack of a supportive environment for adolescent mothers may move beyond their experiences of healthcare facilities and may stem from a poor supportive environment at home. Most adolescent mothers in our sample were single parents (82% of our sample is unmarried), and anecdotal evidence from our FGDs revealed that their pregnancies were rarely supported by family members and parents.

This study provided a quantitative investigation of the causes and covariates of late ANC access among adolescents in metropolitan Cape Town. The data and analysis presented here support the idea that the overall study goal was met.

### Limitations of the study

This study used self-reported data on timing of access to ANC. This measure may suffer from recall and social desirability bias. It would therefore be important in future to obtain clinical estimates of gestation at the time of ANC access. This study analysed data for mothers aged 16–18 years. Antenatal care attendance among mothers younger than 16 years may take place even later in pregnancy. The role of unwanted pregnancies in the timing of ANC access among this group of mothers should be further explored in studies with appropriate ethical design and clearance processes.

Because of an inability to obtain proxy parental consent from the ward councillor for the area in which one of the four facilities were based, this facility had to be dropped from the study. This area was subject to violent service delivery protests at the time the survey was conducted. While adolescents living in this area are likely to be exposed to more everyday incidents of violence, there is insufficient data to support the idea that they would be more socio-economically vulnerable than other adolescents. However, the omission of this facility may have created an inadvertent study bias. We were unable to conduct any supplementary or alternative research in the area because of a lack of permission for data collection in the area.

## Conclusion

Almost two-thirds (66.15%) of pregnant adolescents had their first ANC booking after 3 months of gestation or did not attend ANC at all. Major contributors to delayed care-seeking behaviour include poor pregnancy identification, and a lack of knowledge and information around ANC. Late care-seeking behaviour was also often driven by delayed or deliberate postponement of the appointment. Age and education are protective of health-seeking behaviour, while alcohol consumption is a negative predictor of timing of ANC.

## Appendix 1

**TABLE 1-A1:** Linear probability model results for women who attended antenatal care ≥ 3 months or not at all.

Determinant	Variable	(1)	(2)	(3)	(4)	(5)	(6)	(7)
Did not attend clinic at all	-	0.311***	0.313***	0.299***	0.300***	0.260***	0.270***	0.255***
Unwanted pregnancy	-	-	0.137*	0.118	0.137*	0.145**	0.148**	0.158**
Consumed alcohol	-	-	-	0.158**	0.156**	0.143**	0.150*	0.145**
Employed	-	-	-	0.011	-0.020	-0.011	-0.046	-0.036
Age	Age 16	-	-	Reference	Reference	Reference	Reference	Reference
	Age 17	-	-	0.044	0.047	0.045	0.034	0.031
	Age 18	-	-	-0.070	-0.099	-0.107	-0.111	-0.122
Education	In school	-	-	-	-0.074	-0.068	-0.070	-0.054
	Behind in school	-	-	-	-0.038	-0.040	-0.031	-0.037
Socio-economic status	Quintile 1	-	-	-	-	Reference	Reference	Reference
	Quintile 2	-	-	-	-	-0.160	-0.170*	-0.167**
	Quintile 3	-	-	-	-	-0.107	-0.118	-0.116
	Quintile 4	-	-	-	-	-0.021	-0.021	-0.016
	Quintile 5	-	-	-	-	-0.033	-0.022	-0.020
Black African		-	-	-	-	Reference	Reference	
	Mixed race	-	-	-	-	-	-0.083	-0.087
	Indian	-	-	-	-	-	0.069	0.059
Marital status	Unmarried	-	-	-	-	-	Reference	Reference
	Married	-	-	-	-	-	-	0.014
	Living with partner	-	-	-	-	-	-	0.117
Constant	-	0.536***	0.516***	0.545***	0.610***	0.679***	0.725***	0.711***
*R*-squared	-	0.116	0.133	0.142	0.148	0.163	0.170	0.174
*N*	-	195	195	195	195	195	195	195

* , *p* < 0.1;

** , *p* < 0.05;

*** , *p* < 0.01; bootstrapped standard errors.
